# Pandrug-resistant Acinetobacter Baumannii Infection Identified in a Non-intensive Care Unit Patient: A Case Study

**DOI:** 10.7759/cureus.6321

**Published:** 2019-12-07

**Authors:** Theodore Balfousias, Alexandros Apostolopoulos, Stavros Angelis, Dimitrios Filippou, Spyridon Maris

**Affiliations:** 1 Orthopaedics, General Hospital Hellenic Red Cross Korgialenio Benakio, Athens, GRC; 2 Orthopaedics, East Surrey Hospital Surrey and Sussex Healthcare National Health Service Trust, Redhill, GBR; 3 Surgery, Medical School of National and Kapodistrian University of Athens, Athens, GRC; 4 Orhopaedics, General Hospital Hellenic Red Cross Korgialenio Benakio, Athens, GRC

**Keywords:** acinetobacter baumannii, pandrug-resistant, hospital-acquired infections, resistant strains, antibiotic regimen, antibiotic resistance

## Abstract

Acinetobacter baumannii is a major cause of hospital-acquired infections, particularly in patients treated in intensive care units (ICUs). It can be a causal agent of conditions like pneumonia, bacteremia, meningitis, soft-tissue, and urinary tract infections, and is associated with high mortality rates. We present a case of a 72-year-old male patient treated for fractured neck of femur who went on to develop an infection from a pandrug-resistant Acinetobacter baumannii isolated in blood and urine cultures during his hospitalization in trauma and orthopedic ward. The patient was operated on the second day following his injury with a cephalomedullary nail device and became febrile with rigors on day six. His clinical condition deteriorated over the next days and his inflammatory markers reached a peak value on day 10 post-injury. Acinetobacter baumannii was isolated from blood and urine cultures and a regimen combining rifampicin, tigecycline, and vancomycin in their maximum doses was initiated. The patient was discharged on day 26 post-injury. Before discharge, he had received the above-mentioned intravenous antibiotic regimen for 14 days. He had also been afebrile for six days and undergone three consecutive negative blood culture samples.

## Introduction

Acinetobacter baumannii is a major cause of nosocomial infections, mainly in patients in intensive care units (ICUs). It can be a causal agent for diseases like pneumonia, bacteremia, meningitis, soft-tissue and urinary tract infections, and is associated with high mortality rates [1]. Even though it was considered a pathogen of low virulence in the past, it has recently developed an emerging resistance against many antibiotics. The World Health Association (WHO) has categorized it as one of the bacterial pathogens with disconcerting resistance to antibiotics, under the acronym “ESKAPE” (Enterococcus faecium, Staphylococcus aureus, Klebsiella pneumoniae, Acinetobacter baumannii, Pseudomonas aeruginosa, and Enterobacter) [2]. We present a case of a pandrug-resistant Acinetobacter baumannii isolated in blood and urine cultures in a patient under treatment for a hip fracture while hospitalized in trauma and orthopedic ward.

## Case presentation

A 72-year-old male patient was referred to the emergency department due to an intertrochanteric neck of femur fracture sustained during a car accident. A trauma CT scan excluded any major pelvic and abdominal lesions and also provided a more detailed look at the comminuted extracapsular neck of femur fracture. The patient was on treatment for hypertension and hypercholesterolemia.

The patient was admitted to the trauma and orthopedic department and, after optimization of his clinical parameters and laboratory test values (two units of packed red blood cells and one unit of fresh frozen plasma were required prior to surgery), he underwent closed reduction and internal fixation of his fracture with an intramedullary nail device on day two post-injury. During surgery, one unit of packed red blood cells was administered. The patient was recovering well and one more unit of packed red blood cells was administered on day four post-injury (Hb level: 7.4 g/dL). On day six (fourth postoperative day) the patient complained of rigors and his temperature reached 37.8 °C. Clinical investigation revealed normal vital signs, normal respiratory sounds, and no signs of infection or inflammation of the wound. A urine sample was sent for analysis and culture and sensitivity (C&S), which came back normal. On day seven (fifth postoperative day), the patient’s temperature rose to 38.2° C and blood samples for culturing were collected. Inflammatory markers were elevated [C-Reactive Protein (CRP): 56 mg/L; white blood cell count (WBC): 11,000 μL; erythrocyte sedimentation rate (ESR): 47 mm/hr]. On day 9 (seventh postoperative day), the patient started complaining about weakness, rigors, and dysuria, and his temperature rose to 40 °C. Mean blood pressure was 70/50 mmHg, mean heart rate was 95/min, and the 24-hour urinary output was 2,300 ml. A new urine sample was sent for analysis (positive for urinary tract infection) and C&S, and new blood culture samples were obtained and a chest X-ray was performed. Inflammatory markers on day 10 (eighth postoperative day) were significantly elevated (CRP: 350 mg/L; WBC: 22,000 μL; ESR: 100 mm/hr), and the patient was on the verge of septic shock. After consultation with the infectious disease department, a regimen including rifampicin 600 mg quaque die (QD), colistin 4.5 million international units (IU) bis in die (BID), and tigecycline 100 mg BID was initiated.

On day 12 post-injury, urine and blood cultures isolated pandrug-resistant Acinetobacter baumannii. The patient’s clinical condition remained critical. After further consultation with the infectious disease department, an adjustment was made to the antibiotic regimen, which now included rifampicin 600 mg QD, tigecycline 100 mg, and vancomycin 1,000 mg BID. The patient’s condition slowly showed signs of improvement. On day 16 post-injury, inflammatory markers were improved (Table 1). The patient was afebrile on day 20 post-injury. He was discharged on day 26 post-injury. He had received 14 days of the intravenous antibiotic regimen. He had also been afebrile for six days and undergone three consecutive negative blood culture samples.

**Table 1:** Inflammatory markers on admission and during various stages of hospitalization WBC: white blood cells; CRP: C-reactive protein; ESR: erythrocyte sedimentation rate

	First day of admission	7th day after admission	10th day after admission	16th day after admission (4th day after the final antibiotic regimen)	20th day after admission (8thday after the final antibiotic regimen)	26th day after admission (14thday after the final antibiotic regimen)
WBC (μL)	10,500	11,000	22,000	12,500	11,800	9,800
CRP (mg/L)	22	56	350	114	62	7
ESR (mm/hr)	11	47	100	86	53	25

## Discussion

Acinetobacter baumanii is a strictly aerobic, non-motile, oxidative-negative, Gram-negative bacillus. It has emerged as a major cause of hospital-acquired infections, especially in ICUs. Immunosuppression, prolonged hospitalization, and severe underlying diseases are considered as factors that make patients prone to infection from this pathogen [3]. It colonizes the skin and can be isolated from oropharyngeal and pulmonary secretions of infected patients [4]. Although it was earlier believed to be susceptive to most antibiotics, Acinetobacter baumanii has become a public health concern due to its developing resistance to a wide range of antibiotics [5]. Multidrug- and pandrug-resistant strains have also been isolated.

The incidence of pandrug-resistant Acinetobacter baumannii has recently increased and is associated with a high mortality rate [6]. Assimakopoulos et al. have published a case series regarding the management of pandrug-resistant Acinetobacter baumannii. In 10 patients with ventilator-associated pneumonia, the combination of IV colistin, high-dose IV tigecycline, high-dose IV ampicillin/sulbactam, and inhaled colistin resulted in clinical success in nine patients, and microbiological eradication in seven [7]. Several authors have suggested that pandrug-resistant strains should also receive empirical methicillin-resistant Staphylococcus aureus (MRSA) coverage. They have also described a synergism between colistin and these agents [8,9].

The severity of underlying pathologies is a major risk factor for an infection by a pandrug-resistant strain, and it can be estimated by the acute physiology and chronic health evaluation (APACHE) II score [10]. Patients with prolonged bed stay of more than 30 days, patients with tracheostomy, and patients who have undergone hemodialysis after the placement of a central venous line are also threatened by a pathogen. Moreover, the prior administration of broad-spectrum antibiotics (glycopeptides, carbapenems, piperacillin-tazobactam, fourth-generation cephalosporins) is also considered a risk factor [11-13].

In our case, there were none of the aforementioned risk factors for a pandrug-resistant infection. The patient was not an ICU patient. After isolation of the pathogen and consultation with the hospital's infectious control team, the patient was immediately transferred and isolated in a single room in the infectious disease department ward. Treatment was performed by highly trained nursing staff who followed all precautionary measures. Site cultures were taken from all potential inoculation areas (theatre, orthopedic ward), but no match was identified. No secure speculation as to where the patient was colonized can be attempted.

## Conclusions

Acinetobacter baumanii is an emerging cause of hospital-acquired infections, especially in ICUs. We have presented a case of a 72-year-old male patient treated for a neck of femur fracture who developed a pan drug-resistant Acinetobacter baumannii isolated in blood and urine cultures. There were no risk factors for the development of a pan drug-resistant infection and the patient was never placed in an ICU. No secure speculation as to where the patient was colonized could be attempted. Τhese kind of pandrug-resistant strains in common hospital wards is an alarming phenomenon that should be addressed with extreme caution.
